# Big data and personal information privacy in developing countries: insights from Kenya

**DOI:** 10.3389/fdata.2025.1532362

**Published:** 2025-04-04

**Authors:** Johnson Masinde, Franklin Mugambi, Daniel Wambiri Muthee

**Affiliations:** ^1^Data, Information and Knowledge Management, University of Embu, Embu, Kenya; ^2^Computer and Information Science, Kenyatta University, Nairobi, Kenya

**Keywords:** personal information privacy, developing countries, Kenya, big data, personal data privacy

## Abstract

The present study examined the correlation between big data and personal information privacy in Kenya, a developing nation which has experienced a significant rise in utilization of data in the recent past. The study sought to assess the effectiveness of present data protection laws and policies, highlight challenges that individuals and organizations experience while securing their data, and propose mechanisms to enhance data protection frameworks and raise public awareness of data privacy issues. The study employed a mixed-methods approach, which included a survey of 500 participants, 20 interviews with key stakeholders, and an examination of 50 pertinent documents. Study findings show that the regulatory and legal frameworks though present are not enforced, demonstrating a gap between legislation and implementation. Furthermore, there is a lack of understanding about the risks posed by sharing personal information, and that more public education and awareness activities are required. The findings also demonstrate that while people are prepared to trade their personal information for concrete benefits, they are concerned about how their data is utilized and by whom. The study proposes the establishment of a National Data Literacy Training and Capacity Building Framework (NADACA), that should mandate the training of government officials in best practices for data governance and enforcement mechanisms, educate the public on personal data privacy and relevant laws, and ensure the integration of data literacy into the curriculum, alongside the provision of regular resources and workshops on data literacy. The study has significant implications for policymakers, industry representatives, and civil society organizations in Kenya and globally.

## Introduction

In recent years, the rapid advancement of Fourth Industrial Revolution (4IR) technologies has dramatically accelerated data generation across individual, enterprise, and institutional levels, giving rise to what is now termed “big data”. Big data refers to the innovative approaches that organizations, including government agencies and corporations, employ to combine multiple digital information and use statistical and data mining tools to reveal hidden and unexpected correlations (Rayes and Salam, 2022).

This vast and ever-growing volume of data produced by interconnected devices, social platforms, transactions, and various digital systems offers unprecedented opportunities for insights and innovation (Birch et al., 2021). Harnessing big data allows for more informed decision-making, predictive analytics, and the potential to transform industries by enhancing efficiency, personalization, and competitiveness (Choi and Park, 2022).

While the concept of personal information may initially seem straightforward, emergence of 4IR technologies have significantly blurred its boundaries, making it increasingly complex to define. The rise of Internet of Things (IoT), artificial intelligence, and pervasive data collection tools means that personal information now extends far beyond traditional identifiers like names, phone numbers and addresses (Cheng et al., 2022). Today, seemingly harmless data such as browsing habits, device usage patterns, or even biometric data can be combined to create detailed profiles of individuals (Zhang et al., 2022). This complex web of data points challenges what constitutes personal information, as evolving technologies continuously redefine the depth and scope of what can be tracked, analyzed, and potentially exploited.

The current data privacy practices operate on assumptions that personal data collection, distribution, and lifespan are inherently limited (Ogbuke et al., 2022). However, in the era of the 4IR, these assumptions no longer hold. Personal data including web identifiers, click streams, and PC files are continuously collected by unknown entities, often beyond individuals' awareness or control (Rayes and Salam, 2022). The reality is that once data leave an individual's possession, they take on a life of their own, passing through multiple hands from transaction counterparts to credit agencies, government bodies, and an extensive network of data integrators. In the event of a breach, the information spreads even further to fourth parties, reinforcing the notion that data today has an indefinite lifespan, with no concerted efforts to impose expiration limits (Mulongo et al., 2022).

Consumers often remain unaware of corporate privacy policies and rely on organizations to vouch for the trustworthiness of service providers (Aldboush and Ferdous, 2023). Many do not realize that transaction records are automatically shared with third-party entities, such as credit bureaus, upon completion of a transaction. Once shared, data slip beyond the control of the original owners, and its future use becomes both unknown and unknowable. Additionally, some third-party organizations operate in legally ambiguous spaces, appropriating and exploiting data without explicit consent, leading to unpredictable consequences (Rayes and Salam, 2022).

Government intervention in data usage, whether for national security or other purposes, further complicates the legal landscape (Aldboush and Ferdous, 2023). For instance, the interception of international transactions by the U.S. government via the SWIFT electronic funds transfer network remains a contentious issue. Likewise, the expansion of state surveillance in countries such as China, Russia, the UK, and Australia exemplifies the increasing pre-emption of personal data by governments, raising significant concerns over privacy rights and legal accountability.

Kenya, like many other nations, has experienced a significant expansion in the adoption of 4IR technologies, reshaping how citizens interact with government entities, businesses, and other institutions (Wamsler, 2023). Innovations ranging from mobile banking to e-government services have markedly increased the digital footprint of Kenyan citizens, delivering substantial benefits in efficiency, accessibility, and service delivery (Kimani and Masiga, 2022). This transformation has enhanced financial inclusion and streamlined access to essential services, yet it also presents new challenges, particularly regarding the protection of sensitive personal data. As digital interactions become integral to daily life, there is a heightened need for robust data privacy frameworks that safeguard citizens' information while fostering trust in digital platforms (Chang, 2021). Data protection has become crucial as governments rapidly digitize services, making an online presence almost indispensable for accessing various public and private resources. Consequently, Kenya must carefully balance technological progress with the imperative of strong, adaptive data protection measures to ensure sustainable digital growth.

Data loss is a widespread global phenomenon, with increased frequency in reports of data breaches compromising individuals' personal information held by organizations. As these incidents increase, it becomes evident that managing personal information privacy is no longer solely within an individual's control (Batko and Ślęzak, 2022). The global state of privacy legislation remains fragmented, while some countries have enacted comprehensive privacy laws, others have yet to implement any regulations, and many are still engaged in ongoing legal debates (Floridi and Taddeo, 2018).

The risks of data loss and big data misuse are especially high in Kenya, where data privacy regulations are still relatively new. Article 17 of the International Covenant on civil and Political Rights calls for the state's protection of personal information and privacy. Similarly, the Kenyan Constitution of 2010 recognizes privacy as a basic right. To protect this right, the Data Protection Act of 2019 was passed and went into force in November 2019. This law relates to data controllers and processors, and it defines the parameters for lawful data processing approaches. Its deployment is especially opportune considering the significant expansion of big data as digital technologies become more widely adopted.

Due to limited resources, Kenya's Data Protection Commissioner confronts major hurdles in effectively executing their role. Insufficient funding causes the commission to struggle with implementing its mandates and investing in proper infrastructure to confront evolving data breaches (Kimani and Masiga, 2022). The rapid advancement of technology poses challenges for data protection laws, as these laws frequently struggle to keep up with the changing technological landscape, such as World coin, artificial intelligence, the rise of Internet of Things devices, and social media platforms, which bring about new ways of personal data sharing and processing. The law may not provide guidance on how to address these technological advances.

Earlier studies have investigated consumer privacy concerns and how they affect business performance and regulatory measures (Birch et al., 2021; Kimani and Masiga, 2022). However, there still exist a gap on methodical understanding of how privacy issues occur, notably in the context of big data and the usage of multiple technologies to support big data strategy. To bridge this gap, this study examines the critical tensions between big data and personal information privacy in Kenya. The paper investigates critical concerns of big data and personal information privacy in Kenya, examining the efficacy of current data protection laws and policies. It also delves into the challenges that individuals and organizations confront in protecting their data privacy in an ever-complex technological environment. Furthermore, the study makes recommendations for strengthening data protection regimes and raising public awareness about privacy issues in Kenya. By focusing on Kenya, this study seeks to broaden the understanding of the complex interplay between big data and personal information privacy in emerging economies. It also aims to contribute to policy discussions on aligning the potential benefits of big data with the need to safeguard individual confidentiality.

## Literature

Big data, as a concept, has been in existence for over two decades, first introduced by Elliott and Soifer (2022). Initially, it referred to the massive volumes of scientific data, but its definition has since evolved. IBM characterizes big data by three key attributes: volume, variety, and velocity. Volume signifies the vast amounts of data generated from multiple sources, including mobile applications, smart grids, and social media platforms such as Facebook (Naeem et al., 2022).

In recent years, big data has emerged as a dynamic field, where technological advancements enable innovative methods to process massive datasets in near real-time, sourced from diverse origins (Elliott and Soifer, 2022). It supports “big” analytics, which unlock new opportunities by repurposing data, identifying correlations, and uncovering novel applications beyond traditional structured databases. These analytics extend across various domains, from academic research to e-commerce and service delivery in everyday life (Naeem et al., 2022).

Despite its benefits, big data also raises critical concerns, particularly regarding privacy and data protection. While it enhances emergency response, security, healthcare, and climate change predictions, it also presents challenges. As Batko and Ślęzak (2022) highlights, the sheer number of data sources makes it difficult for individuals to maintain control over their personal information. Many users remain unaware of how their data is processed or transferred between systems, creating significant challenges in ensuring transparency and accountability, especially when multiple stakeholders are involved.

Another key issue is data reusability. Big data analytics often leverage existing datasets, either independently or in combination with other sources, beyond their original purpose of collection. The scalability of storage infrastructure allows for continuous data accumulation, with the expectation that new insights will emerge over time (Ogbuke et al., 2022). For instance, mobile applications that track fitness or health metrics such as heart rate or dietary habits collect user data that could be valuable to insurance companies, sports centers, or dietary consultants, potentially leading to targeted services or offers.

Additionally, integrating multiple datasets can generate new insights, but it also introduces privacy risks. The combination of different data sources can inadvertently reveal patterns linked to individuals, even when the original datasets are considered non-personal (Rayes and Salam, 2022). Cheng et al. (2022) note that sophisticated analytics can infer personal details by merging datasets, leading to unintended processing of personal information. Similarly, re-identification techniques using anonymized data can expose sensitive details, posing risks to individuals' privacy. In some cases, this could result in serious consequences, such as reputational harm or threats to personal safety due to the disclosure of confidential information (Birch et al., 2021).

### Emerging technologies and data privacy issues

In the era of Big Data, the vast availability of data resources and powerful analytic techniques have rendered traditional privacy protection methods ineffective (Muthee, 2023). As data collection and processing become more pervasive, maintaining conventional perceptions and attitudes toward privacy is increasingly challenging. The long-standing notion of data privacy as a simple interaction between consumers and businesses is no longer sufficient in the Fourth Industrial Revolution (4IR) (Batko and Ślęzak, 2022). While 4IR technologies promise to enhance various aspects of daily life, they simultaneously expose personal information on an unprecedented scale, often without adequate safeguards.

The rapid advancement of 4IR technologies has enabled the extensive exploitation of personal data, surpassing any level of data collection seen before (Payton and Claypoole, 2023). While many view data as a valuable asset, a series of high-profile data breaches such as the Cambridge Analytica scandal and Equifax data breach have raised global concerns about privacy and data security (Aldboush and Ferdous, 2023). These incidents underscore the urgent need to reassess and strengthen personal data protection frameworks.

Artificial Intelligence (AI) stands at the forefront of privacy challenges, with several studies highlighting its role in eroding personal privacy (Aldboush and Ferdous, 2023; Muthee, 2023; Naeem et al., 2022). AI-powered facial recognition, predictive analytics, and surveillance technologies have been criticized for enabling mass data collection without adequate consent or oversight (Elliott and Soifer, 2022). For instance, AI-driven deepfake technology has been exploited for identity theft and misinformation, while algorithmic biases in data processing have led to ethical concerns regarding discrimination and surveillance.

The Internet of Things (IoT) further complicates privacy risks by facilitating seamless data exchange between billions of connected devices, from smartphones and wearables to smart homes and autonomous vehicles (Rayes and Salam, 2022). With machine learning capabilities, these devices can autonomously process and share data, often without direct user control. However, the IoT's inherently complex and decentralized nature creates significant challenges in securing the vast amounts of data flowing through interconnected networks. This complexity increases vulnerabilities, making personal data susceptible to unauthorized access and cyber threats.

Additionally, cloud computing environments have revolutionized data storage by transferring vast amounts of personal information to virtual platforms (Abdulsalam and Hedabou, 2021). While offering convenience and scalability, cloud storage exposes individuals to privacy breaches, as sensitive details of their personal lives such as financial records, health data, and communication logs are stored on third-party servers, making them attractive targets for cyberattacks (Batko and Ślęzak, 2022).

### Big data and privacy concerns in Kenya

Multiple studies have highlighted the possible threats to personal information privacy associated with the use of big data in developing nations such as Kenya. For instance, Kimani and Masiga (2022) found that many Kenyan citizens were unaware of the extent to which their personal information was collected and processed by various organizations, and expressed concern about the possibility of their information being used for unintended purposes or being accessed by unauthorized parties. Similarly, the Kenya Human Rights Commission (2017) reported a lack of effective legislative frameworks and enforcement mechanisms to protect citizens' data privacy rights in Kenya, notably in the context of government monitoring and data breaches.

In light of these concerns, the data protection landscape in Kenya has seen substantial transformations in recent times. In 2019, the country passed the Data Protection Act, which provided a regulatory framework for personal data collection, processing, and sharing. However, challenges persist in implementing and enforcing the Act, particularly in the face of the quickly expanding digital landscape. For example, Konde-Lule and Mabawonku (2020) identified the need for increased public knowledge of data privacy rights and the risks of big data misuse, as well as the significance of providing training and support to data protection officers and other stakeholders. Kamau and Wanjohi (2019) investigated the use of big data analytics in Kenya's insurance industry and discovered that, while it had the potential to improve risk assessment and pricing, there were concerns about data accuracy and reliability, as well as the possibility of discrimination and bias in algorithmic decision-making.

Otieno et al. (2021) investigated the function of social media platforms in the acquisition and usage of personal data in Kenya. According to the survey, many Kenyans use social media platforms for a variety of objectives, including socializing, entertainment, and information sharing, but they are often unaware of how much personal information these platforms gather and analyze. The survey also found that Kenyans are unaware of their data privacy rights, and that more public education and awareness-raising efforts are needed to empower citizens to take control of their personal data. Kimani and Masiga (2022) investigated the use of big data analytics in Kenya's financial sector. The study discovered that, while big data has the potential to improve credit scoring and risk management in the sector, there were issues about the data's integrity and completeness, as well as the possibility of discrimination and prejudice in decision making. The report also underlined the need for a more complete legislative and regulatory framework to oversee big data use in the financial sector, as well as to protect personal data privacy and security.

Njoroge et al. (2020) investigated the obstacles associated with adopting data protection rules and legislation in Kenya. The study discovered that, while the country had made progress in enacting data protection laws, significant challenges remained in implementing and enforcing these laws, particularly due to regulatory agencies' limited capacity and a lack of awareness and compliance among businesses and other organizations. According to the report, tackling these issues would necessitate a multifaceted approach that includes more public education and awareness-raising campaigns, strengthened enforcement mechanisms, and greater stakeholder participation.

Finally, Muthoni et al. (2019) investigated the ethical implications of using big data in Kenya's healthcare sector. The study emphasized the potential benefits of big data for patient care and disease surveillance, but it also raised concerns about the privacy and security of personal health information, as well as the possibility of discrimination and prejudice in algorithmic decision-making. The study advised that healthcare providers and policymakers collaborate to set ethical principles and best practices for the use of big data in healthcare, as well as ensure that patients are fully informed about the dangers and benefits of sharing their personal health information.

The literature on big data and personal information privacy in developing nations, such as Kenya, emphasizes the importance of a complete and coordinated strategy to data protection that takes into account these countries' unique issues (Esteban-Navarro et al., 2020). As big data becomes more critical in economic development, public services, and decision-making, it is critical that policymakers, businesses, and other stakeholders collaborate to protect individuals' privacy rights and reduce the potential risks of big data misuse.

### Theoretical framework

This study was guided by the Privacy Calculus Theory and the Surveillance Capitalism theory, which collectively provide a comprehensive lens for understanding the implications of big data on personal data privacy. The Privacy Calculus Theory suggests that individuals engage in a cost-benefit analysis when sharing personal data, weighing the perceived benefits of digital services against potential privacy risks. However, in the Big Data era, where data collection is often opaque and automated, individuals may be unable to make fully informed privacy decisions (Martin and Zimmermann, 2024). The study used this theory to explore how users perceive privacy risks and benefits in the evolving technological landscape and whether traditional privacy protection mechanisms remain effective.

Additionally, the Surveillance Capitalism Framework, as introduced by Zuboff (2023), highlights how corporations and governments leverage data extraction as a core economic model. This framework explains how personal data is commodified, analyzed, and monetized at an unprecedented scale, often without users' explicit consent or awareness. By applying this theory, the research assessed the extent to which individuals' autonomy over their data is diminished in the digital economy and whether regulatory measures are sufficient in mitigating these concerns.

### Conceptual framework

**Figure 1:**
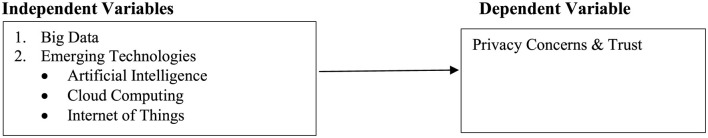
Conceptual framework.

This framework represents how Big Data directly influence user behavior in terms of privacy concerns (Figure 1).

## Methodology

The study employed a mixed-methods approach, capturing and analyzing data from both qualitative and quantitative sources. The approach enabled a more thorough comprehension of the subject and aided in the triangulation of results from various sources (Gregar, 2023; Masinde et al., 2024). The study's target audience comprised Kenya's 23,848,956 adults (Kenya Human Rights Commission, 2017) who are directly impacted by big data and personal information privacy issues. The survey was conducted with a representative sample of 500 people from 10 counties in Kenya. A purposive sampling technique was used to select 20 significant stakeholders for interviews who included government officials, legal experts, and industry representatives. The document study included an examination of 50 key regulations, legal documents, industry reports, and academic literature on big data and personal information privacy in Kenya.

### Data collection

The study employed a number of data collection methods:

a) Surveys: A structured questionnaire was administered to 500 respondents through online platforms and in-person distribution. Krejcie and Morgan (2020) assert that a sample size of 500 is often deemed acceptable for larger populations and is frequently employed in social science research. This is because it ensures high precision and accuracy. It also achieves an optimal balance between statistical power and practical considerations such as resources and logistics (Wambiri and Masinde, 2019). The survey employed a stratified random sampling technique to ensure representation across different demographic groups. The questionnaire covered areas such as data privacy awareness, experiences with data breaches, trust in digital platforms, and perceptions of government's data-handling practices. Likert-scale and multiple-choice questions were included to quantify attitudes and trends. Respondents included professionals, business owners, students, and general citizens from urban and rural areas. In addition, the researchers ensured a complete response study. This was achieved through rigorous follow ups and reminders.
b) Interviews: In-depth, semi-structured interviews were conducted with 20 key stakeholders to gain qualitative insights into policy implementation, challenges in regulating big data, and potential solutions for enhancing personal data privacy. According to Bricman et al. (2023), a sample size of 10–20 is generally deemed sufficient for conducting interviews since it provides for an in-depth examination of participants' experiences, viewpoints, and beliefs. Purposive sampling was used to identify individuals with expertise or influence in big data policies and privacy frameworks. These included government officials, legal experts, industry representatives and civil society advocates. Interviews were conducted in person or via virtual platforms, recorded (with consent), and transcribed for thematic analysis.
c) Document Review: 50 key documents which included regulations, legal documents, industry reports, and academic literature were reviewed to gain a thorough understanding of the existing regulations, frameworks, best practices as well as the challenges and opportunities presented by big data. A systematic review process, guided by PRISMA, ensured the selection of 50 relevant documents from authoritative sources such as government archives, legal repositories, and academic databases. According to Bowen (2009), a sample size of 50 documents is commonly considered acceptable for document analysis since it permits a comprehensive examination and yields sufficient data for analysis and interpretation. The documents were selected based on relevance, credibility, and objectivity (Guest et al., 2023).

### Reliability and validity

To enhance the reliability and validity, the survey instrument was pre-tested with a small group (*n* = 30) to refine question clarity and consistency. Triangulation was employed by cross-referencing findings from surveys, interviews, and document reviews to strengthen the study's credibility and depth.

### Data analysis

The survey data was examined using descriptive statistics to uncover patterns and trends in respondents' attitudes and behaviors toward personal information privacy. The statistical tool, Statistical Package for the Social Sciences (SPSS) was employed to generate frequency distributions, mean scores, and correlations between variables related to privacy concerns, demographic factors, and trust levels.

Thematic analysis was employed to analyze interviews to discern pertinent themes and patterns to the study. Key themes were identified through coding, categorization, and pattern recognition to gain qualitative insights into policy implementation, challenges in regulating big data, and potential solutions for enhancing personal data privacy.

Content analysis was employed to examine the documents to uncover important themes and patterns in Kenya's personal information privacy policies and regulatory frameworks. Key provisions, recurring themes, and gaps in existing privacy policies were highlighted, enabling a comparative assessment of regulatory approaches.

The study faced multiple challenges during the processes of data collection and analysis. These included limited data literacy and privacy awareness among certain respondents, which affected the quality of survey responses and necessitated further clarifications. Furthermore, logistical constraints, including internet connectivity issues and scheduling conflicts, hindered the participation of interviewees, especially in remote areas. Certain stakeholders also exhibited reluctance to engage in discussions regarding privacy issues, influenced by regulatory sensitivities and confidentiality concerns. Additionally, during the document analysis phase, accessing specific government and corporate privacy policies was challenging due to restrictions on classified information. Despite these challenges, methodological rigor and triangulation strategies enhanced the study's credibility and depth (Resnik, 2021).

### Ethical considerations

Ethics in research warrant that no individual suffers the harmful consequence of research activities (Masinde and Sanya, 2022). Consequently, the Investigator took time to inform respondents of the nature and aim of the research before requesting their consent.

## Findings and discussions

### Demographic characteristics

A varied and extensive group was examined to guarantee thorough and in-depth insights into how different population segments in Kenya interact with big data and personal information privacy. The demographic findings are presented in the table below (Table 1).

**Table 1:** Participants' demographic characteristics.

**Characteristic**	**Category**	**Percentage**
Gender	Male	53%
	Female	47%
Age group	18–25	29%
	26–35	37%
	36–45	23%
	46 and above	11%
Level of education	High school	12%
	Undergraduate	56%
	Postgraduate	32%
Sector of employment	Private	58%
	Public	33%
	Non-profit	9%

Study's findings demonstrate that the gender distribution is approximately balanced, with males constituting 53% and females 47% of the population. Prior research shows that gender affects data privacy concerns. An investigation by Munung et al. (2024) revealed that males are more inclined to disclosing their data in return for tangible rewards, but their female counterparts exhibited greater caution over data sharing. Furthermore, a study by Netshakhuma (2020), that investigated personal information in Sub-Saharan Africa revealed that women with limited data literacy levels were more inclined to disclosing their personal information in return for convenience. These findings suggest that future research on personal data privacy and awareness should address gender-specific concerns. Targeted digital literacy awareness programs could also help address the gap, given the nearly equal gender distribution.

The findings also imply that the majority of participants were in the age groups of 26–35, followed by 18–25, suggesting that younger individuals are generally more familiar with data applications, including electronic commerce, social media, and banking services. However, a study by Laibuta (2024) found that young individuals exhibited a greater tendency to share personal information in exchange for tangible benefits. These findings suggest that data protection authorities should prioritize data literacy programs for young adults.

Moreover, the study's findings indicate that a significant majority of participants (56%) possessed a bachelor's degree, suggesting a greater likelihood of their comprehension of big data and personal data privacy issues. Prior studies have demonstrated a positive correlation between data literacy comprehension and educational attainment (Sutherland, 2018; Laibuta, 2024).

A significant proportion of young individuals in the demographics suggests elevated interactions with big data and privacy data, which also signifies increased risks to data privacy. The limited participation of the older generation highlights the need for enhanced data literacy programs and the strengthening of the current data protection act to align with the General Data Protection Regulation (GDPR) and other robust personal data regulations (Mokhtari and Malekinezhad, 2022). Businesses and government entities should be encouraged to adopt effective data management practices.

The study revealed a myriad of insights into big data and personal information privacy in Kenya. The findings are presented as per the themes below:

### Effectiveness of present data protection laws and policies

The study shows that a majority of the respondents (68%) lacked confidence in governmental institutions regarding the confidentiality of personal information. Many respondents expressed concerns about the government 's ability to protect their data, with some citing examples where their information had been mishandled or misused by government agencies. However, the interview findings presented a contrasting perspective, with trust in government institutions emerging as a strong theme. Several interviewees (Codes 1, 3, 4, 7, 12, 15, 17, 18, and 19) expressed confidence in the government's ability to safeguard citizens' private data, acknowledging that while a few rogue officers might have misused data for personal gain, these incidents were exceptions. The findings are corroborated by Agbozo et al. (2018), who established that individuals in Sub-Saharan Africa exhibit a lack of trust in government's capacity to safeguard their data, due to inadequate enforcement mechanisms and the absence of well-defined frameworks for privacy data protection. The survey further showed that government agencies primarily focus on data harvesting for surveillance and administrative purposes, while neglecting the safeguarding, which has led to recurrent data breaches and misuse.

The relevant government authorities should ensure enforcement of the set laws and regulations governing personal data and investigate data breaches. The government should also ensure transparency in the way personal information is handled from the time it is collected to when it is disposed. Government agencies should be audited to ensure they are complying with the laws and regulations governing personal information.

Second, the investigation revealed that Kenya has established multiple legal and regulatory frameworks that pertain to data privacy including the International Covenant on Civil and Political Rights (Article 17), which advocates for privacy and the protection of personal information by the State. Similarly, the Constitution of Kenya (2010) guarantees the right to privacy as a fundamental right. To further protect individual privacy, the Data Protection Act, 2019, was enacted and came into effect the same year. However, the study identified a significant challenge in implementing these laws. Only 27% of surveyed respondents were familiar with the Data Protection Act of 2019. These findings align with Sutherland (2018), who identified a significant gap between legislation and implementation of data privacy laws and regulations in emerging economies. The study indicates that although most emerging economies have established laws and regulations about personal data privacy, government officials, including enforcement officers and the general public lack awareness of these laws and their respective roles and responsibilities. The study revealed that this resulted in ongoing abuse and misuse of personal information, as individuals were unaware of their rights, and specific government or enforcement officers lacked knowledge of the relevant laws, leading to weak or nonexistent implementation.

Moreover, the government should implement public data literacy programs to educate the public on the laws and regulations related to personal information and the methods for safeguarding their data against misuse. The government should also consider developing online tutorials or conducting regular workshops on safeguarding personal information.

### Challenges individuals and organizations experience while securing their data

The findings identified lack of awareness of the risks associated with sharing personal information online as a major challenge. A considerable majority (63%) of the surveyed participants were unaware of the risks associated with sharing personal information online. Interviews further corroborated these findings, revealing a persistent theme of lack of awareness among the general population. As one respondent (Code H17) stated:

“Many Kenyans, particularly the youth, are inclined to sharing their entire lives online without contemplating the associated risks.”

Another interviewee (Code 3) observed:

“Many of us agree to the terms of service on various platforms without conceptualizing how our data will be used.”

Document analysis of 50 industry reports and academic sources corroborated these findings, revealing that 62% of the literature shows that people are largely oblivious to the risks associated with sharing personal information.

These findings are particularly concerning due to the increasing overreliance on big data applications such as social media, electronic commerce, and financial institutions. The findings may be attributed to insufficient data literacy programs, inadequate enforcement of data regulations by the responsible authorities, and a lack of concern regarding the sharing of personal information. These findings align with Sutherland (2019), who observed that the general population in sub-Saharan Africa largely lacks awareness of the risks related to sharing personal information, such as names, phone numbers, and locations, online. Furthermore, many individuals utilize digital platforms and share their personal information without significant concern for the associated risks. The research also indicated that a majority of public servants improperly managed personal information, due to insufficient data literacy and inadequate enforcement mechanisms. In Kenya, the Data Protection Commissioner plays a critical role in raising public awareness of data privacy and enforcing the Data Protection Act of 2019. Nonetheless, study findings have also shown that the Commissioner faces significant challenges due to insufficient resources, which impede the effective execution of its mandate (Prinsloo and Kaliisa, 2022). The lack of enough resources impairs the commission's ability to promote public awareness, enforce data protection laws, and invest in the necessary infrastructure to address the increasingly complex issues related to data breaches. These findings demonstrate the need for the government and relevant entities to implement national data literacy programs. The government should engage various media platforms, including television, radio, and social media, to ensure that the data literacy programs are accessible throughout all regions of Kenya, including remote areas.

Second, the study identified ignorance of the legislation that governed personal information as another challenge. Fifty-seven percentage of the surveyed participants were ignorant of the legislation that governed personal information. These findings were collaborated by the Interviews that also revealed a recurring theme of ignorance about Kenya's specific data protection legislation. These findings align with Alunge (2019) who established that individuals, including government officials, are ignorant of personal data privacy laws and regulations, resulting in inadequate enforcement and misuse of personal information. The research identified the challenge as stemming from insufficient data literacy programs.

The study findings reveal a complex and somewhat contradictory perception of government institutions' role in personal information privacy in Kenya. This divergence between public perception and expert opinion suggests a need for greater transparency and communication from government agencies to build trust in their data protection practices. The government should implement mandatory data literacy training programs to educate officials on enforcement mechanisms and best practices in data governance. Third, the study identified economic pressures as another challenge. Survey results indicated that a significant proportion of Kenyans (59%) are willing to share their personal information in exchange for incentives or rewards, such as discounts or complementary products. However, only a small minority (16%) felt comfortable sharing their data with third-party advertisers. The interview findings emphasized this pattern, suggesting that many Kenyans prioritize incentives over data privacy, potentially due to economic pressures. For example, one respondent (Code 16) mentioned that:

“Observe how hundreds of Kenyans converged on the World coin Orb crypto project to have their irises scanned for $50; many were indifferent regarding the utilization of their data.”

Document analysis yielded limited findings on this issue, with only one document indicating that some websites offered discounts in return for personal referrals, forcing customers to provide personal details of others, such as names, email addresses, and phone contacts. The study's findings reveal a complex dynamic between the desire for incentives and the concern for data privacy among Kenyans. These findings imply that although rewards may encourage data sharing, there is a need for greater awareness about the potential risks associated with such practices. The findings align with Zwitter and Gstrein (2020), who observed that individuals in developing nations exhibit a propensity to share their data, due to a lack of awareness about data privacy risks, coupled with economic challenges and potential benefits such as financial rewards, discounts, incentives, and convenience. Prinsloo and Kaliisa (2022) further asserts that a significant number of individuals expressed a readiness to share their data, grounded in the trust they place in institutions, as they are confident that their information would not be exploited, dismissing concerns related to identity theft, surveillance, and the commercialization of personal data.

The propensity of individuals to share personal information for incentives underscores the need for the government to establish a national data literacy initiative that emphasizes the risks associated with such disclosures. The government should establish stringent data protection protocols and require organizations that gather personal information to furnish individuals with explicit consent forms, ensuring comprehension of data collection, storage, and management practices.

### Mechanisms to enhance data protection and awareness of data privacy issues

Based on the findings, this study advocates for the establishment of a National Data Literacy Training and Capacity Building Framework (NADACABU), that should:

a) Mandate the training of government officials in best practices for data governance and enforcement mechanisms;
b) Educate the public on personal data privacy and relevant laws and;
c) Ensure the integration of data literacy into the curriculum, alongside;
d) Provision of regular resources and workshops on data literacy.

### Implications

#### Theoretical implications

The findings of this study offer theoretical implications for Privacy Calculus Theory and the Surveillance Capitalism Framework which guided the study. The findings suggest that while individuals engage in a rational cost-benefit analysis when disclosing personal data, as outlined by Privacy Calculus Theory, their decision-making is often affected by low literacy levels in dangers of sharing personal information and insufficient regulatory knowledge. The finding that 63% of respondents were unaware of the risks associated with sharing personal information online and that only 27% were familiar with the Data Protection Act of 2019 underscores a fundamental limitation of the theory: it assumes that individuals make informed choices. However, in the context of big data many users are not well-informed which laces them at a disadvantage, thereby challenging the effectiveness of the traditional privacy trade-off model. This finding suggests that Privacy Calculus Theory need to evolve to account for the structural barriers such as opaque data practices and inadequate digital literacy skills that prevent users from accurately assessing privacy risks.

Moreover, the study reinforces and extends the Surveillance Capitalism Framework by demonstrating how individuals' autonomy over their personal data is systematically undermined. The widespread distrust in governmental institutions (68%) regarding data confidentiality aligns with Zuboff's (2023) argument that surveillance mechanisms operate with minimal accountability. The improper handling of personal information by public servants further illustrates how regulatory frameworks fail to curb exploitative data practices, increasing concerns over privacy erosion. The study findings suggest that the Surveillance Capitalism Framework should integrate a more localized perspective, recognizing how socio-political and regulatory inefficiencies contribute to the persistence of digital exploitation in specific national contexts. Collectively, the study's insights call for a reconsideration of privacy theories to better capture the realities of digital governance, power asymmetries, and the role of institutional trust in shaping individuals' privacy behaviors.

### Practical significance

The study's findings yield two significant implications for enhancing data privacy practices and policies in Kenya. The prevalent readiness of Kenyans to divulge personal information for rewards, despite minimal trust in data protection, underscores an urgent need for public education initiatives. These initiatives should seek to enhance knowledge on the dangers of data sharing and assist individuals in making more informed choices.

To address the Data Protection Commissioner's financial limitations, the Kenyan government should provide appropriate resources, allowing the Commissioner to conduct successful data privacy awareness programs.

Second, the general distrust in government institutions' ability to secure personal information, combined with varying levels of confidence among stakeholders, shows that government agencies should improve transparency and communication. Establishing public trust necessitates that these institutions not only secure data efficiently but also actively interact with the public, addressing concerns and demonstrating their fidelity to data protection.

### Limitations of the study

Even though the study offers significant insights into big data and personal information privacy in Kenya; it encountered several limitations. The survey depended on self-reported data, which may have been biased; the sample size of 20 interviewees, while knowledgeable, may not have encompassed all viewpoints on data privacy; and the document review was restricted to publicly accessible resources, potentially omitting confidential government or corporate data policies.

### Conclusions

Study's findings highlight key concerns and opportunities for data privacy in Kenya. A considerable segment of the populace is still oblivious to the dangers of divulging personal information, with multitudes prepared to trade their data for incentives despite potential repercussions. This behavior underscores the need for comprehensive public education programs to enhance understanding of data privacy and enable individuals to make sound choices. The disconnect between popular skepticism in governmental institutions and the confidence demonstrated by key stakeholders implies that more transparency and participation from government agencies is necessary for establishing and maintaining public trust in data protection initiatives.

Furthermore, the study demonstrates that although Kenya has instituted legislative frameworks like the Data Protection Act of 2019, lack of awareness among the public, industry stakeholders, and government officials present a significant hurdle to successful implementation. The Data Protection Commissioner's office's limited resources exacerbate the challenge. To resolve these challenges, it is imperative for the Kenyan government to allocate adequate resources and support to the Data Protection Commissioner, to enable the office execute its mandate and at the same time safeguard the privacy of individuals in an increasingly digital landscape

In summary, the findings of the study advocates for the establishment of a National Data Literacy Training and Capacity Building Framework (NADACABU) that should mandate the training of government officials in best practices for data governance and enforcement mechanisms, educate the public on personal data privacy and relevant laws and ensure the integration of data literacy into the curriculum, alongside provision of regular resources and workshops on data literacy.

## Data Availability

The raw data supporting the conclusions of this article will be made available by the authors, without undue reservation.
